# Synthesis, Purification, and Characterization of Carbon Dots from Non-Activated and Activated Pyrolytic Carbon Black

**DOI:** 10.3390/nano12030298

**Published:** 2022-01-18

**Authors:** Reyna Berenice González-González, Lucy Teresa González, Marc Madou, César Leyva-Porras, Sergio Omar Martinez-Chapa, Alberto Mendoza

**Affiliations:** 1 Escuela de Ingeniería y Ciencias, Tecnologico de Monterrey, Ave. Eugenio Garza Sada 2501, Monterrey 64849, Mexico; reyna.g@tec.mx (R.B.G.-G.); lucy.gonzalez@tec.mx (L.T.G.); smart@tec.mx (S.O.M.-C.); 2 Department of Mechanical and Aerospace Engineering, University of California Irvine, Engineering Gateway 4200, Irvine, CA 92697, USA; mmadou@uci.edu; 3 Laboratorio Nacional de Nanotecnología (Nanotech), Centro de Investigación en Materiales Avanzados (CIMAV), Miguel de Cervantes No. 120, Chihuahua 31136, Mexico; cesar.leyva@cimav.edu.mx

**Keywords:** carbon nanoparticles, top-down synthesis, chemical activation, waste valorization

## Abstract

In this work, carbon dots were created from activated and non-activated pyrolytic carbon black obtained from waste tires, which were then chemically oxidized with HNO_3_. The effects caused to the carbon dot properties were analyzed in detail through characterization techniques such as ion chromatography; UV–visible, Fourier-transform infrared spectroscopy (FTIR), X-ray photoelectron spectroscopy (XPS), and Raman spectroscopy; ζ potential; transmission electron microscopy (TEM); and spectrofluorometry. The presence of functional groups on the surface of all carbon dots was revealed by UV–visible, FTIR, XPS, and Raman spectra. The higher oxidation degrees of carbon dots from activated precursors compared to those from nonactivated precursors resulted in differences in photoluminescence (PL) properties such as bathochromic shift, lower intensity, and excitation-dependent behavior. The results demonstrate that the use of an activating agent in the recovery of pyrolytic carbon black resulted in carbon dots with different PL properties. In addition, a dialysis methodology is proposed to overcome purification obstacles, finding that 360 h were required to obtain pure carbon dots synthesized by a chemical oxidation method.

## 1. Introduction

Carbon dots (CDs), a type of carbon nanomaterial with a size below 10 nm and a quasi-spherical shape, were discovered in 2004 by Xu et al. [1]. Their intriguing properties, such as biocompatibility and fluorescence, have made them important in a variety of fields [2,3,4,5,6,7]. They can be synthesized in two ways: bottom-up and top-down. The bottom-up approach uses physical and chemical forces to organize atoms or molecules into the desired nanostructure, whereas the top-down approach involves reducing the size of the precursor until it reaches the desired nanostructure [8].

The many potential applications of CDs have led to intense research of environmentally friendly and less expensive synthesis methods [9]. In the case of top-down approaches, the precursors used for nanomaterial synthesis, which are waste carbon-rich materials, have an important role as an excellent option for the conversion of low-value materials into high-value products [10].

In this manner, waste tires could be considered an ideal precursor to produce CDs because nearly 76% of their composition are carbon-based materials [11], and using a precursor with a low economic value reduces the cost of the process. The feasibility of CD synthesis from waste tires, on the other hand, is based on the fact that most organic compounds containing C, H, and O, where H and O exist in a form that allows dehydration, can be used for CD synthesis [12]. Furthermore, existing commercial waste tire pyrolysis processes could be supplemented with an additional route of high-value products (e.g., CDs), or carbon dots from waste tires could be processed as an additional production line in a conventional pyrolysis plant.

The conceived application of CDs in next-generation devices would require very small quantities of ultrapure CDs with precisely controllable size, shape, composition, and surface features. Any wet chemical synthesis method is unlikely to produce such high-quality CDs. Such CDs must be manufactured using sophisticated deposition or epitaxial growth techniques. However, some applications, such as carbon dot-based bulk nanocomposites for thermoelectric material [13], require a large number of CDs, preferably with some variation in size, shape, and surface features. The method described in this paper is a good and low-cost alternative for these applications.

Pyrolysis is a technique that can be used to convert waste into nanomaterials [10]. Through this process, waste tires are thermally decomposed into char, liquid, and syngas [14,15,16]. The pyrolytic char is a carbon-rich source composed of carbon black and inorganic substances used during tire manufacturing [15,17,18], whose characteristics such as surface area, chemical surface composition, and pore development can be enhanced through activation processes [19,20]. During chemical activation, the carbon material is impregnated with a chemical activation agent (acid or alkali) before carbonization [19,21]. A variety of chemical agents have been used, including KOH as a high surface area promoter [19,21,22,23] and H_2_SO_4_ as a porosity controller [24].

The variety of existing carbon precursors and different synthesis methods has led to CDs with diverse properties and structures [25], making it necessary to study the effects associated with each variation. The effect of the synthesis method on the properties of the CDs has thus been investigated; Crista et al. [26] discovered differences in nitrogen doping between the different bottom-up strategies (hydrothermal, microwave assisted, and calcination). Similarly, the effects of varying synthesis process parameters such as reaction time [27] and the use of different precursors [28] have provided useful information for understanding CD properties. The modification of the carbon precursor by an activation process may contribute to the understanding of the properties of CDs.

To properly analyze CDs, a purification process before characterization is needed, since byproducts (frequently formed during CDs synthesis) can modify CD fluorescence [29,30,31]. Essner et al. [30] demonstrated in this context that impurities should be removed in order to obtain reliable results on the properties of CDs. Furthermore, they examined a sample of over 550 reports and discovered that more than half of them used insufficient purification processes [30]. Some researchers investigated the required dialysis time to ensure CDs purification [29,30], whereas others used column chromatography for the same purpose [31]. However, these studies were performed on CDs synthesized by bottom-up pathways. An advantage of this synthesis approach is the production of CDs with a higher purity [32]. Therefore, the evaluation of the purification process of CDs synthesized by a chemical oxidation process (a top-down pathway) is needed.

In this study, CDs are synthesized from activated and non-activated pyrolytic carbon black from waste tire pyrolysis processes. The effects of the chemical composition of the precursor on the properties of CDs are investigated. Furthermore, the CDs dialysis purification process is examined to determine the time required for complete purification.

## 2. Materials and Methods

### 2.1. Carbon Dots Synthesis

**Figure 1:**
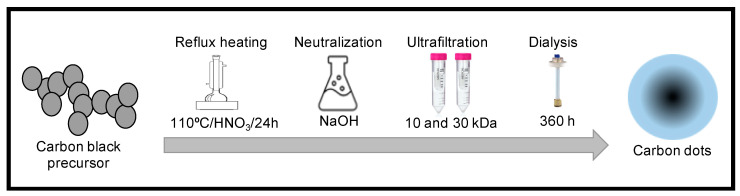
Chemical oxidation methodology followed for the synthesis of carbon dots from carbon black and activated carbon black as precursors.

Four different samples of carbon black were used as precursors for carbon dot synthesis using an oxidation method, followed by neutralization, ultrafiltration, and dialysis (Figure 1).

Carbon black precursors were recovered by four different pyrolysis/activation processes: (1) pyrolysis, (2) H_2_SO_4_ activation and pyrolysis, (3) KOH activation and pyrolysis, and (4) H_2_SO_4_ activation, KOH activation, and pyrolysis. Carbon black was recovered from waste tire powder with a particle size of 75–125 µm obtained from LT Lehigh Technologies (Tucker, GA, USA). The detailed methodology is reported in [33]. In brief, when chemical activation (either H_2_SO_4_ or KOH) was used, waste tire powder was impregnated at 80 °C with an activating agent/tire ratio of 4 before being filtered and dried. The activation temperature, weight ratio, and activation agents were chosen based on optimal values reported in the literature for improving the properties of carbon black [19,21,33]. The pyrolysis process was performed on a Lindberg Blue M tubular muffle (Thermo Scientific, Watertown, WI, USA) with a heating rate of 10 ᵒC/min until reaching 950 °C with a nitrogen flow of 200 mL/min. 

The recovered carbon black from each process was collected and used as a CD precursor with an adapted version of the chemical oxidation technique documented by Dong et al. [34]. Reflux heating of the carbon black precursors was performed at 110 °C with 6M HNO_3_ for 24 h with continuous stirring, then NaOH was used for neutralization. Finally, purification was performed by ultrafiltration and dialysis for 360 h using a Spectra-Por^®^ Float-A-Lyzer^®^ G2 of 10 mL with a membrane of 3.5–5 kDa. To ensure complete carbon dot purification, a Dionex ICS-1600 Ion Chromatography System was used after 48 h, 192 h, 240 h, and 360 h of dialysis. 

In the following sections of the manuscript, the synthesized carbon dots are identified as follows: carbon dots synthesized from carbon black as “Cd.CB”, carbon dots from H_2_SO_4_-activated carbon black as “Cd.H_2_SO_4_”, carbon dots synthesized from KOH-activated carbon black as “Cd.KOH”, and carbon dots from H_2_SO_4_ and KOH-activated carbon black as Cd.H_2_SO_4_, and KOH.s (4) (i.e., H_2_SO_4_ activation, followed by pyrolysis and KOH activation) as “CB.H_2_SO_4_ + KOH”.

### 2.2. Carbon Dots Characterization

The optical properties of carbon dots were analyzed by UV–visible spectroscopy; this technique is useful to observe the region of absorption, which on carbon dots is typically in the UV region. On the other hand, some properties such as stability, biocompatibility, and photoluminescence are highly dependent on the presence of functional groups on their surface; therefore, characterization techniques such as FTIR, XPS, Raman, and ζ potential were performed. The analysis of the particle size, size distribution, and morphology was performed using images obtained by scanning electron microscopy (SEM) and transmission electron microscopy (TEM). Finally, a detailed analysis of the photoluminescence was performed, which is of great interest since many of its applications are based on these properties.

Optical properties of diluted carbon dot suspension were analyzed with PerkinElmer UV/Vis Lambda 365 equipment. The absorbance measurements were performed in the 200–800 nm wavelength range. A Frontier FT-IR Spectrometer (PerkinElmer, Waltham, MA, USA) with the Universal ATR sampling accessory was used to analyze functional surface groups. The measurements were performed in the transmission range of 400 cm^−1^ to 4000 cm^−1^ with 64 scans. XPS analysis was performed with a Thermo Scientific ESCALAB 250Xi spectrometer (East Grinstead, West Sussex, UK) equipped with 8 Channeltron detectors. AlK_α_ radiation (1486.68 eV) was used as a source of ionizing radiation. The survey spectra were measured at the 1100 eV pass energy with a resolution of 1 eV, 100 ms, and 150 eV pass energy. High-resolution spectra were collected at 0.1 eV, 100 ms, and 20 eV pass energy. The analysis was performed under ultrahigh vacuum (10^−10^ Torr), and signals were assigned using the NIST database.

Carbon structure was studied by Raman spectroscopy using LabRAM HR Evolution (Horiba Scientific, Jobin Yvon, Longjumeau, France) equipment with a 532 nm laser as an excitation source. Raman scattered light was detected by a CCD camera operating at 220 K. An Olympus microscope SLMPN 50×/0.35 NA was used to focus the laser on the sample. Raman signals were acquired at 25%, 50%, and 100% of the beam intensity. Band intensity, band position, and area were obtained using a Lorentz curve fitting procedure on the normalized Raman spectra.

To analyze the surface charge of the carbon dots, the ζ potential was measured by Phase-analysis light scattering (PALS) with a NanoBrook 90Plus PALS Brookhaven instrument performing 10 measurements per sample and using the Smoluchowski equation to convert the electrophoretic mobility of the particles obtained by the instrument into the ζ potential. Carbon dots nanoparticles were observed using Transmission electron microscopy (TEM). Bright-field (BF) images were acquired in a transmission electron microscope Hitachi HT7700 operated at 100 kV. Carbon dots samples were dispersed in distilled water with the aid of an ultrasound bath. A drop of the dispersed solution was placed on a 300-mesh copper grid covered with a lacey carbon film. With a diffraction aperture of 500 nm in diameter, selected area electron diffraction (SAED) patterns were obtained. The fluorescence was measured with a Horiba FluoroMax-4 Spectrofluorometer with an excitation range of 340 nm to 543 nm and an emission range of 370 nm to 750 nm.

## 3. Results and Discussion

### 3.1. Carbon Dot Purification

**Figure 2:**
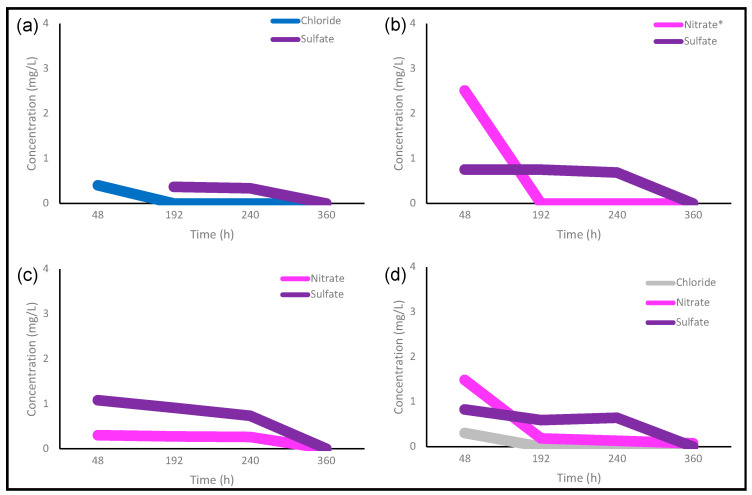
Ion chromatogram of (**a**) Cd.CB, (**b**) Cd.H_2_SO_4_, (**c**) Cd.KOH and (**d**) Cd.H_2_SO_4_ + KOH at different dialysis times. Note: * Nitrate concentration in panel b was divided by four for comparison purposes.

The formation of undesirable byproducts, unreacted precursors, or impurities is almost inevitable during the synthesis of carbon dots. Thus, synthesized carbon dots were purified through dialysis before their analysis. Figure 2 shows the ion chromatography results from carbon dots after 48 h, 192 h, 240 h, and 360 h of dialysis. The presence of chloride, nitrate, and sulfate was observed, though these were not detected after 360 h of dialysis. The presence of nitrate salts is caused by the neutralization of HNO_3_ with NaOH. The presence of sulfates may not be due to H_2_SO_4_ activation of the precursor because CDs from nonactivated carbon black had a low concentration of sulfates. Sulfates may be formed during chemical oxidation with the precursor, which reported a sulfur content between 1.4% and 4.5% [33]. The low concentration of chloride might be due to the dilution preparations of the required chemical agents and from the double-distilled water used during dialysis. 

Chen et al. [29] reported that it takes around 120 h to completely purify CDs synthesized from citric acid, which is much longer than the regular dialysis time used in CD studies, suggesting also that the purification is frequently insufficient [29]. Ion chromatography revealed that it took up to 360 h to purify the carbon dots in our study. Purification times obtained here are longer than those reported for carbon dots synthesized using bottom-up synthesis methods by Chen et al. [29], who reported ∼120 h to completely purify CDs. Synthesis methods belonging to the top-down approach lead to a relatively high yield of products; however, the purity of products is typically higher when the bottom-up synthesis approach is performed [32]. Moreover, if carbon dots are prepared from biomass or waste materials instead of pure chemicals, numerous impurities can be formed. In this manner, the synthesis of carbon dots from biomass or waste precursors is still challenging due to non-standard synthesis methods and the presence of impurities, which may lead to a longer time for a proper purification.

### 3.2. Carbon Dot Characterization

Pyrolytic carbon black recovered by waste tire pyrolysis was used as the starting material. Four different processes were performed to modify the characteristics of the recovered carbon black: (1) pyrolysis, (2) H_2_SO_4_ activation and pyrolysis, (3) KOH activation and pyrolysis, and (4) H_2_SO_4_ activation, KOH activation, and pyrolysis. The complete characterization of the carbon black recovered from each process is presented in our previous research [33]. The differences presented in their elemental composition, surface area, and Raman spectra (I_D_/I_G_ ratio), obtained by an Organic Elemental CHNS-O Analyzer Flash 2000 (Thermo Scientific^TM^, Waltham, MA, USA), an Autosorb IQ TPX Quantachrome gas adsorption analyzer (Boynton Beach, FL, USA) using the BET method, and a LabRAM HR Evolution (Horiba, Jobin Yvon, Longjumeau, France), respectively, are presented in Table 1. The chemical activations (H_2_SO_4_, KOH, and H_2_SO_4_ + KOH) caused an increase in the oxygen content and surface area compared to the nonactivated carbon black.

**Figure 3:**
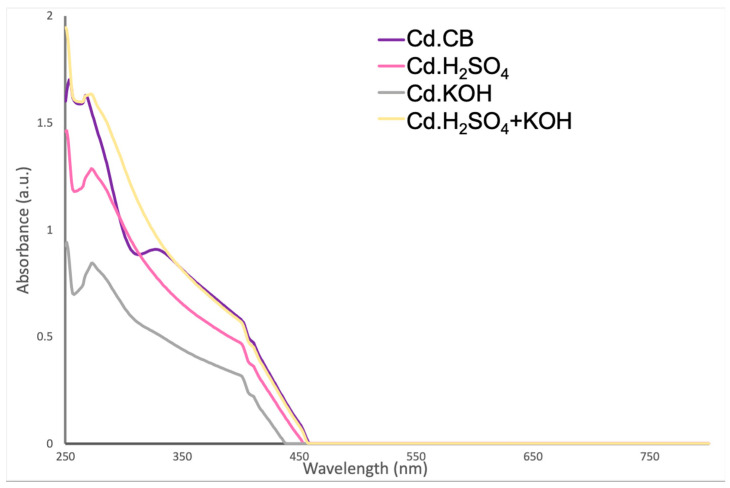
UV–visible spectra of carbon dots.

To analyze the optical properties of carbon dots, UV–visible absorption was measured. Figure 3 shows that carbon dot samples did not present strong differences in the spectra. All samples showed broad adsorption that extended to the visible region, with peaks associated with the carbonic core center at 255 nm and 272 nm [6,35], indicating extended conjugation in the carbon dot structure [6,36]. The adsorption peak at 255 nm is specifically ascribed to the π-π* transition of carbon–carbon double bond (C=C) and the peak at 272 nm to the π-π* transition of carbon–nitrogen double bond (C=N) [37,38]. On the other hand, the absorption peak at 401 nm was related to the surface of the carbon dots [6,35], specifically to the n-π* transition of C=N [39]. An additional peak around 340 nm was mainly observed in Cd.CB, which was attributed to n-π* transition bands on carbon dots demonstrating the presence of doping elements [40].

**Figure 4:**
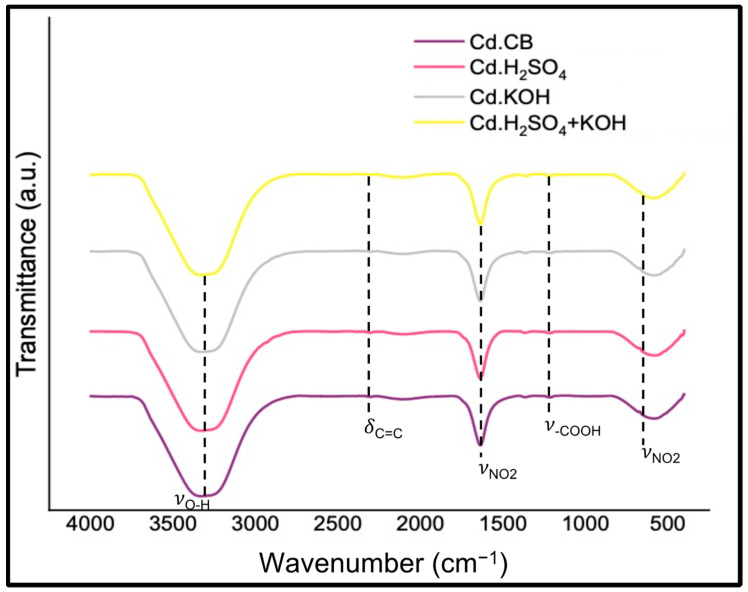
FTIR spectra of carbon dots.

Functional groups in synthesized carbon dots were analyzed by FTIR, XPS, Raman, and ζ potential. In the FTIR spectra presented in Figure 4, a strong broad peak approximately at 3361 cm^−1^ associated with O−H stretching vibration [41,42,43,44,45,46,47] is observed, suggesting the existence of carboxyl and hydroxyl groups resulting in the hydrophilic behavior of the carbon dots samples. The peak at 2100 cm^−1^ is associated with the bending vibration of C=C from aromatic structure [48], while the peak at 1377 cm^−1^ is attributed to the stretching vibration of −COOH [43]. The presence of nitro (NO_2_) groups was confirmed with two peaks approximately at 1639 cm^−1^ and 600 cm^−1^, which are assigned to the symmetric stretching and asymmetric stretching of nitro groups, respectively [49]. FTIR results suggest the presence of carboxyl (COOH), hydroxyl (OH), and nitro (NO_2_) functional groups on the carbon surface introduced during chemical oxidation with HNO_3_ [50,51]. According to Datsyuk et al. [50], HNO_3_−treated carbon material had the highest increase in oxygen-containing groups when compared to other oxidants, which is consistent with other studies [52]. Nitrogen can be introduced through a reaction similar to benzene nitration, producing highly reactive nitronium ion [53,54]. The nitrogen on the surface of CDs may be in the form of nitro groups, as observed in FTIR spectra and literature [51,53,54,55].

**Figure 5:**
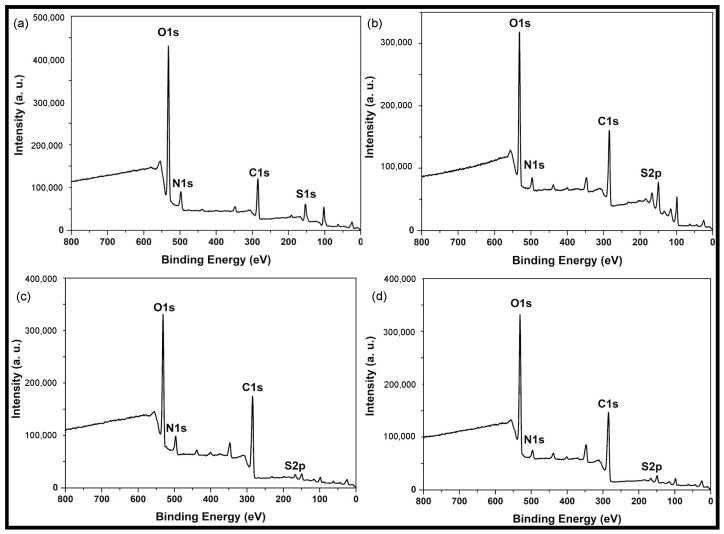
XPS survey spectrum of (**a**) Cd.CB, (**b**) Cd.H_2_SO_4_, (**c**) Cd.KOH, and (**d**) Cd.H_2_SO_4_ + KOH.

XPS was measured to analyze the different types of functional groups on the carbon dots surface. A similar chemical structure was observed in the different carbon dots samples (Figure 5). Four predominant peaks were found: C1s, O1s, N1s, and S2p.

**Figure 6:**
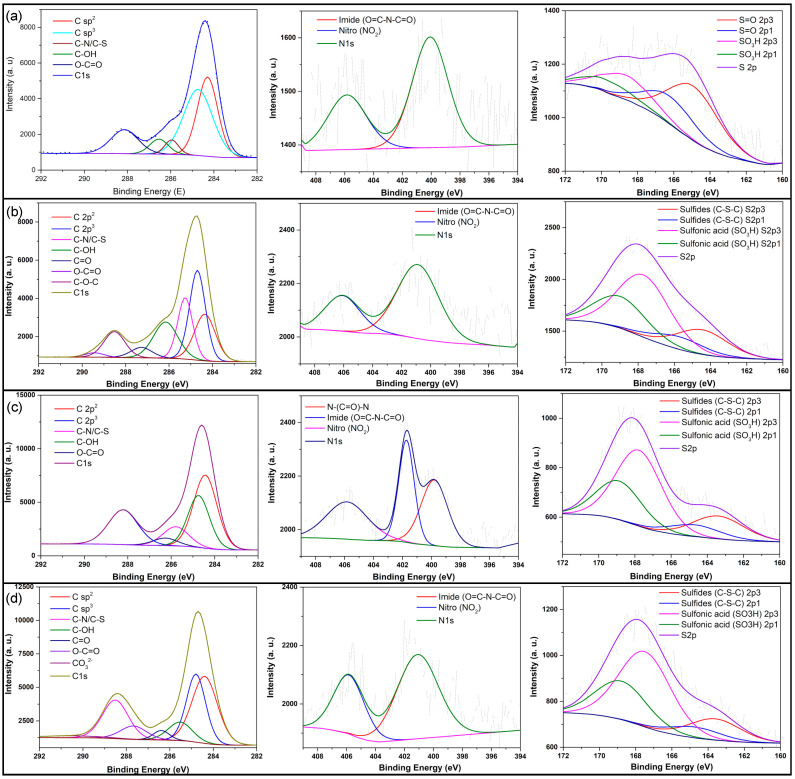
High-resolution XPS spectra of C1s, N1s, and S2p of (**a**) Cd.CB, (**b**) Cd.H_2_SO_4_, (**c**) Cd.KOH, and (**d**) Cd.H_2_SO_4_ + KOH.

C1s, N1s, and S2p peaks were deconvoluted to identify their components (Figure 6). C1s from samples Cd.CB and Cd.KOH was deconvoluted into C sp^2^ (at ~284.29 eV), C sp^3^ (at ~284.73 eV), C−N/C−S (at ~285.96 eV), C−OH (at ~286.54 eV), and O−C=O (at ~288.14 eV). Two additional components were found in Cd.H_2_SO_4_: at 287.83 eV attributed to C=O and C−O−C at 287.10 eV. Similarly, in sample Cd.H_2_SO_4_ + KOH was observed additional peaks at 287.67 eV and 290.61 eV attributed to C=O and CO_3_^2–^, respectively, indicating an even higher oxidation degree in this sample. The atomic percentages of C1s, N1s, and O1s varied as can be seen in Table 2. The sum of the atomic percentage of the oxygen-containing groups in C1s on the carbon dots from activated precursors was in the range of 26.23–31.96%, while in Cd.CB the atomic percentage was 20.21%. The increase in this value suggests a higher oxidation degree in carbon dots prepared from activated precursors. This is also supported by a decrease in hydroxyl (C−OH) and an increase in O−C−O because hydroxyl groups are formed at lower oxidation levels and then converted into epoxy groups as the oxidation level increases [56]. On the other hand, the atomic percentage of N1s and S2p increased on carbon dots from activated precursors, implying that surface functional groups were better attached during chemical oxidation when the precursor was previously activated. The XPS results are in agreement with the FTIR results, suggesting the presence of nitrogen-, oxygen-, and sulfur-containing groups on the carbon dot surface.

**Figure 7:**
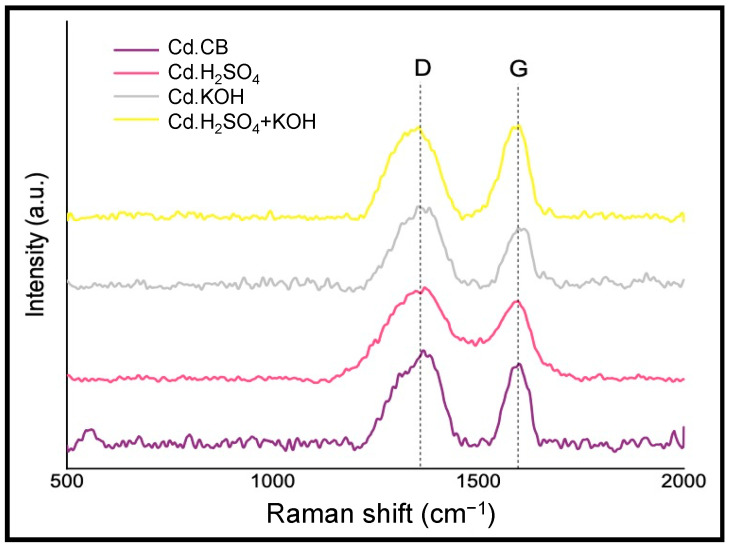
Raman spectra of carbon dots.

Results from Raman spectroscopy are presented in Table 3 and Figure 7. The D band at approximately 1350 cm^−1^ is attributed to the functional groups on the carbon dots surface and indicates hydrocarbon or aliphatic moieties connected to the graphitic structure [11,57]. The G band, which appears around 1580 cm^−1^, corresponds to in-place vibrations and shows the presence of small graphitic planes or microcrystallites. The Raman spectra (Figure 7) obtained from all of the carbon dots samples show a high degree of surface functionalization, which is consistent with the findings of Wu et al. [58], who reported that defects on graphene quantum dots during chemical passivation cause an increase in the D band.

Carbon dots from activated precursors, except for Cd.H_2_SO_4_ + KOH with an I_D_/I_G_ of 0.99, presented an increase in their I_D_/I_G_ (Cd.H_2_SO_4_ with an I_D_/I_G_ of 1.30 and Cd.KOH with an I_D_/I_G_ of 1.38) compared to the I_D_/I_G_ of 1.13 obtained from Cd.CB. This suggests a higher introduction of functional groups in the activated precursors. In addition, all carbon dot samples exhibited an increase in I_D_/I_G_ compared with their respective precursor, showing the effect of the increasing D band due to the oxygen-containing groups on the surface of carbon dots.

The ζ potential values presented in Table 4 were measured to obtain the surface charge of carbon dots and therefore the presence of functional groups. The low absolute value of ζ potential (less than 25 mV) in all carbon dot samples indicates their proclivity to aggregate due to their low physical stability. Cd.CB demonstrated superior stability with the highest absolute value. It should be noted that ζ potential was measured after months of carbon dot synthesis, which likely affected the stability of the carbon dots. However, it is common to find in literature carbon dots with a low absolute ζ potential value, with some successful applications [59,60,61] reporting good stability regardless of the low absolute value. Among these, Radhakrishan et al. [60] evaluated the ζ potential before and after a passivation process, finding a decrease from −18.6 mV to −4.06 when glycine was used as the surface passivizing agent. Despite the low absolute value, over a month, carbon dots exhibited a homogeneous phase with no precipitate formation, and the carbon dots were successfully validated as an effective fluorescent sensor for the detection of metallic ions from aqueous solutions [60].

The negative charge observed in our study indicates a dense electron cloud concentrating on the carbon dots [62], attributed to carboxyl, hydroxyl, or carbonyl groups in the nanoparticle surface [63]. Carbon dots from activated precursors (Cd.H_2_SO_4_, Cd.KOH, and Cd.H_2_SO_4_ + KOH) showed a less negative ζ potential from −11.61 mV to −5.20 mV, −4.22 mV and −1.28 mV, respectively, compared with Cd.CB. The slight increase in the band associated with C=O observed in UV–visible spectra suggests that the increase in negative surface charge on Cd.CB is due to a higher content of electron-withdrawing oxygen-containing groups on its surface.

In addition, a Shapiro–Wilk normality test on the residuals (*p*-value = 0.4791) and Levene’s test (*p*-value = 0.1390) for homogeneity of variance were performed to verify the assumptions of normal distribution and homogeneity of variance, which were fulfilled by the data. The significance of differences in ζ potential values between carbon dots samples was then determined with an ANOVA test. Because all of the process parameters were under control, the differences observed were solely due to the precursor’s activation. A significance value (*p*-value < 0.05) for the interaction of the two factors (presence of H_2_SO_4_ activation and presence of KOH activation in the precursor) was obtained; therefore, a Tukey’s test was performed to identify these differences. Tukey’s tests showed that ζ potential of Cd.H_2_SO_4_ and Cd.KOH are not statistically different, while Cd.CB and Cd.H_2_SO_4_ + KOH are statistically different from each other and compared with the other carbon dots samples. These findings support differences in the surface negative charge of carbon dots associated with functional groups: carbon dots synthesized from nonactivated carbon black had better suspension stability, whereas carbon dots synthesized from activated precursors had a lower negative ζ potential.

**Figure 8:**
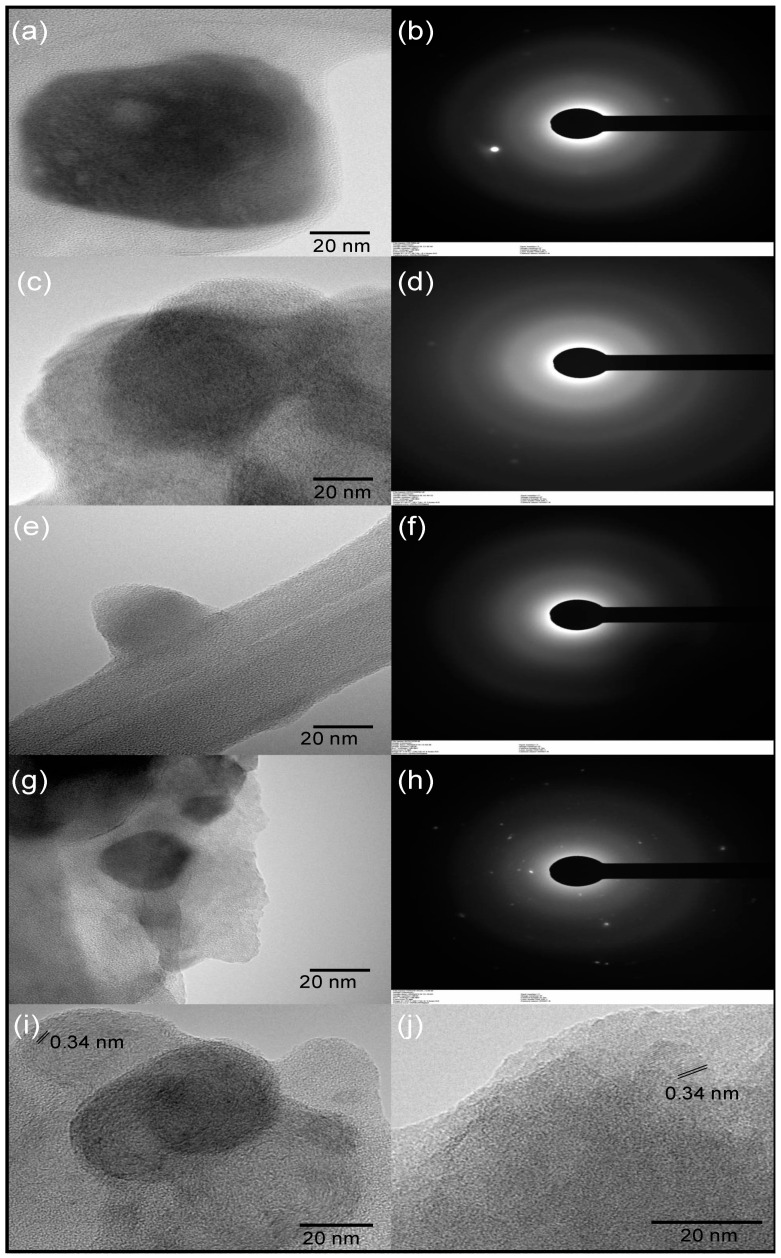
TEM images of (**a**) Cd.CB, (**c**) Cd.H_2_SO_4_, (**e**) Cd.KOH, and (**g**) Cd.H_2_SO_4_ + KOH. Selected area electron diffraction (SAED) pattern of (**b**) Cd.CB, (**d**) Cd.H_2_SO_4_, (**f**) Cd.KOH and (**h**) Cd.H_2_SO_4_ + KOH. TEM images showing lattice fringes of (**i**) Cd.H_2_SO_4_ and (**j**) Cd.H_2_SO_4_ + KOH.

To obtain images of the carbon dots on the nanometer scale, transmission electron microscopy (TEM) was performed. Figure 8 shows bright-field transmission electron microscopy (BF-TEM) micrographs of CDs samples. Overall, spherical-shaped nanoparticles with sizes in the range of 10–50 nm were observed. TEM images of CDs with particle sizes bigger than 10 nm have been also reported in the literature [64,65], which have been mainly attributed to agglomeration. Due to the small size of carbon dots, they tend to agglomerate in the solid state. Agglomeration of the nanoparticles was observed in all samples, which was expected given the ζ potential obtained in the range of −1.28 to −11.61 mV. In addition, aggregation may be also caused by the drying process during sample preparation for TEM analysis [59]. The SAED revealed a diffuse ring diffraction pattern, which is indicative of short-range order or an amorphous microstructure, implying that the samples are mostly amorphous. However, in Cd.H_2_SO_4_ and Cd.H_2_SO_4_ + KOH (Figure 8i,j) some crystalline areas with interlayer space of 0.34 nm were observed. A larger interlayer space than that for crystalline graphite (0.335 nm) and no stacking order indicates a turbostratic graphite structure [66]. This observation suggested that the H_2_SO_4_−activated precursor produces carbon dots with some degree of order in the carbon structure. The KOH-activated precursor, on the other hand, had no crystalline areas, indicating that the amorphous carbon had not graphitized. In summary, the acid activation method produced more graphitized domains than the basic activation method [67], but all of the samples were a random mix of crystalline, amorphous, quasi 2D, quasi 0D, quasi 1Dm, and arbitrary shaped C islands with surface functionalization.

**Figure 9:**
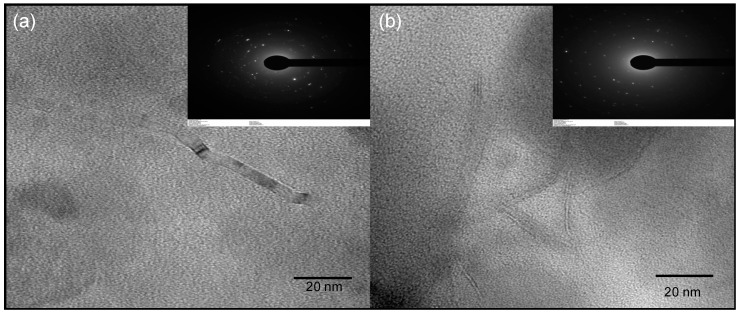
High-magnification BF-TEM micrographs showing structures associated with graphite and graphene, respectively in (**a**) Cd.CB and (**b**) Cd.H_2_SO_4_. The inset figures show the corresponding SAED pattern.

In addition to the spherical carbon dots nanoparticles, other irregular shape carbon particles were observed in TEM micrographs (Figure 9). Sample Cd.CB showed the presence of graphite-like particles, while graphene platelets were observed in samples prepared from Cd.H_2_SO_4_-activated precursors. Different diffracting crystalline planes were visible in the inset SAED patterns in Figure 9a,b, indicating an ordered microstructure. The presence of graphite in the Cd.CB may be due to unreacted precursor residues that could be converted into carbon dots with a longer reaction time. Other reports have found particles that are significantly smaller than the starting material but larger than carbon dots, despite filtration and dialysis [27]. The presence of graphene platelets in the samples from the activated precursors may be attributed to the occurrence of enhanced oxidation. Oxidation is widely used during the exfoliation process of graphite in the synthesis of graphene platelets, forming graphite oxide as intermediate products [68]. The oxygen-containing functional groups in graphite oxide have been shown to induce local strain and facilitate graphene monolayer exfoliation [56,68,69]. The formation of epoxide groups at higher oxidation levels influences the increase in interlayer spacing and exfoliation into graphene oxide [56]; these functional groups were visible in XPS spectra of H_2_SO_4_−activated precursors. Therefore, we suggest that the higher surface area and the presence of reactive functional groups on the activated precursors may enhance the oxidation process facilitating the formation of graphene.

### 3.3. Photoluminescence (PL)

**Figure 10:**
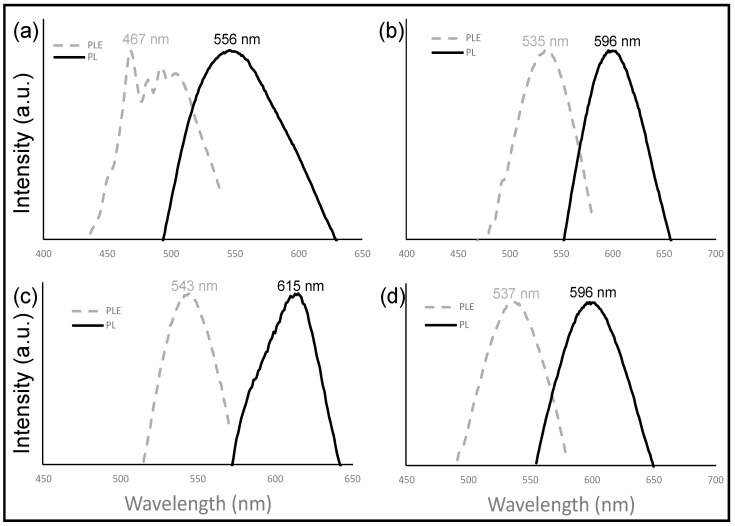
Photoluminescence (PL) and photoluminescence excitation (PLE) spectra of (**a**) Cd.CB, (**b**) Cd.H_2_SO_4_, (**c**) Cd.KOH, and (**d**) Cd.H_2_SO_4_ + KOH.

**Figure 11:**
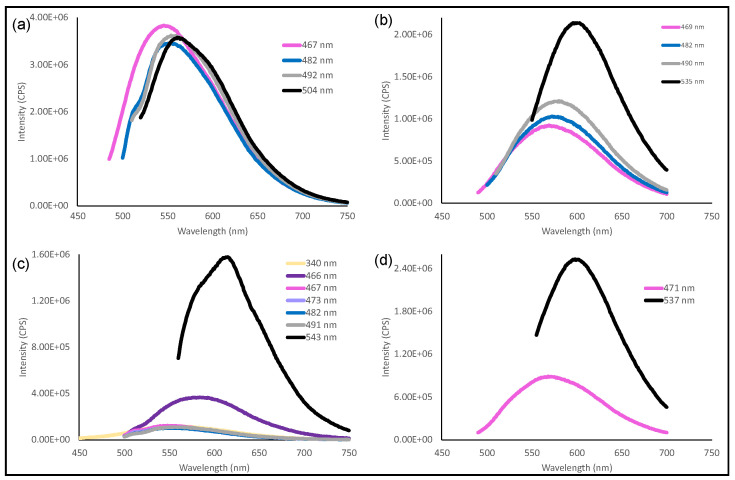
PL spectra at different wavelengths of (**a**) Cd.CB, (**b**) Cd.H_2_SO_4_, (**c**) Cd.KOH, and (**d**) Cd.H_2_SO_4_ + KOH.

The photoluminescence (PL) of carbon dots is of great interest since many of its applications are based on these properties. Although the mechanism of PL from carbon dots has been extensively studied, the origin of fluorescence is still unknown [2,25]. The results of the PL analysis (Figure 10 and Figure 11) revealed differences between the carbon dot samples depending on whether or not their precursor was activated. In comparison to the non-activated sample (Cd.CB), carbon dots from activated precursors showed an obvious bathochromic shift in their PL spectra; Cd.CB had a higher PL intensity; Cd.CB had an excitation-independent behavior, whereas Cd.H_2_SO_4_, Cd.KOH, and Cd.H_2_SO_4_ + KOH had an excitation-dependent behavior. All of these differences in the PL properties are analyzed in detail in this study.

It has been reported that carbon dots from pyrolytic carbon black presented a PL peak at 415 nm at the maximum excitation wavelength (380 nm) [70]. As can be seen in Figure 10, in our study, a bathochromic shift (shift to longer wavelengths) was observed in all carbon dot samples compared to those reported in the literature. This result could be associated with a higher oxidation degree [71,72].

The PL shift was even higher in the carbon dots from activated precursors (Cd.CB < Cd.H_2_SO_4_ < Cd.H_2_SO_4_ + KOH < Cd.KOH). A hypsochromic or bathochromic shift is caused by a bandgap modification [73,74,75,76]. A hypsochromic PL shift is observed with a widening of the bandgap, while the opposite (a bathochromic shift) occurs by narrowing the bandgap [73]. This bandgap is highly dependent on the nanoparticle size and the surface functionalization. The electron delocalization on the carbon structure and electron confinement in the carbon dots causes the bandgap modification caused by the carbon dot’s size [71]. Some researchers have shown that the size of carbon dots affects the bandgap and thus the PL emission [77,78]. The bandgap, on the other hand, is strongly influenced by surface functionalization with oxygen-containing groups: as the oxygen content increases, the bandgap decreases [79]. Some authors have reported that surface oxidation creates surface defects [76], which serve as capture centers for excitons. Further, the bathochromic shift is caused by the radiation from the recombination of trapped excitons [80]. Therefore, it is well accepted that a higher oxidation degree causes a bathochromic shift [71,72,75,79], while a higher surface oxidation and the creation of surface defects are achieved with the use of strong acids [50,81], such as the HNO_3_ used in this study.

In this context, the observed bathochromic shift in the PL spectra of carbon dots from activated precursors (Cd.H_2_SO_4_, Cd.KOH, and Cd.H_2_SO_4_ + KOH) may be caused by a higher oxidation degree than that presented in Cd.CB. These findings are consistent with XPS and Raman results, which showed that carbon dots from activated samples have a higher functionalization. Carbon dots were made by chemical oxidation with HNO_3_, which oxidizes the starting material while also introducing oxygen and nitrogen-containing groups to the surface of the carbon dots. [51,75]. We suggest that functional groups were more easily introduced in the carbon surface when the precursor was previously treated with acid or alkali (activated carbon). Abdel-Nasser and El-Hendawy [51], observed an increase in the oxygen, hydrogen, and nitrogen content when oxidation with HNO_3_ was performed on activated carbon compared with the corresponding non-oxidized char. Other researchers [82,83] found similar results, attributing the higher functionalization by wet oxidation to a larger surface area with more developed textures on the activated carbons. A greater surface area provides a better precursor–HNO_3_ contact, thus enhancing the reactions and introducing more functional groups.

A higher oxidation degree on carbon dots from activated precursors was also demonstrated with the results provided by XPS and Raman spectroscopy [56]. Besides the oxidation degree effect, the presence of nitro groups may induce an additional bathochromic shift on the PL because of the electron-accepting effect from the nitro functional group [75,84,85].

Carbon dots from activated precursors (Cd.H_2_SO_4_, Cd.KOH, and Cd.H_2_SO_4_ + KOH) showed an excitation-dependent behavior. Figure 11b–d show that the abundant functional groups on the surface of carbon dots from activated precursors, as well as the resulting surface defects, may play a role in the excitation-dependent behavior. The heterogeneity of carbon dots [86], as a result of the introduction of surface defects by functional groups with various energy levels [87,88], has been reported to be the source of the excitation-dependent behavior. In particular, oxygen-containing groups have a strong effect on the creation of energy gaps that result in a tunable PL [89]. This tunable emission is important in understanding the electronic states of carbon dots and for various applications, such as biosensing, allowing simultaneous monitoring of various analytes and processes [90] and optoelectrical applications [91]. On the other hand, the emission behavior observed on the carbon dots from the nonactivated precursor (Cd.CB) might be optimal for applications such as fluorescence ink and as a fluorescent sensor for the detection of metallic ions in aqueous solution [86].

Regarding PL intensity, the Cd.CB sample exhibited a different behavior to carbon dots from activated precursors (Figure 11). Carbon dots from activated precursors showed a greater intensity as the excitation wavelength increased, with a maximum value in the range of 535–543 nm; the opposite was observed in Cd.CB, with a maximum PL intensity of 467 nm. Moreover, the PL intensity of Cd.CB is considerably greater than any of the other carbon dot samples (Figure 11a). It has been reported that the oxidation degree of the carbon dots, in addition to the bathochromic shift, has an effect on emission intensity [72]. This effect is due to the surface traps acting as nonradiative recombination centers, which can decrease the PL intensity [92,93]. In addition, the presence of electron-withdrawing groups such as nitro groups causes a decrease in the PL intensity [94]. Therefore, the synthesized carbon dots from activated precursors are rich in oxygen-containing groups and nitrogen-containing groups on their surface as demonstrated by XPS and Raman techniques, which causes its hydrophilicity, negative ζ potential, bathochromic shift, excitation-dependent behavior, and PL intensity decrease.

## 4. Conclusions

In this study, the dialysis purification process was carefully analyzed by ion chromatography, concluding that 360 h were required to completely purify the carbon dots synthesized from pyrolytic carbon black from waste tires by a chemical oxidation method. It was shown that carbon dots could be made from carbon black and activated carbon black. However, whether the precursor was activated or not, some differences in their PL properties were discovered: the region of the electromagnetic spectrum where the PL emission occurred, the intensity of the PL emission, and the excitation-dependent behavior. In addition, some differences in their carbon core were observed; carbon dots synthesized from the H_2_SO_4_−activated precursor exhibited a higher crystallinity (graphitized domains) than carbon dots from non-activated precursor and basic-activated precursor.

The obtained ζ potential, UV–visible, FTIR, and Raman spectra indicated the presence of functional groups such as carboxyl, hydroxyl, and nitro groups in the four synthesized carbon dots. However, carbon dots from activated precursors presented an increase in the ID/IG, suggesting a higher content of surface functional groups. The increase in the functional groups and the high oxidation degree caused a bathochromic shift in their PL spectra with an excitation-dependent behavior, contrary to the behavior observed in Cd.CB (carbon dots from non-activated precursor). Although the PL mechanism is still unknown, it is reasonable to believe that the chemical composition and surface morphology of the precursor influence the optical properties of carbon dots due to the better attachment of surface functional groups during chemical oxidation with HNO_3_. Understanding the observed differences in the optical properties of carbon dots is essential for suggesting their possible applications.

## Figures and Tables

**Table 1:** Characterization of recovered carbon black used as precursor.

	Carbon Black	H_2_SO_4_-Activated Carbon Black	KOH-Activated Carbon Black	H_2_SO_4_ + KOH-Activated Carbon Black
Nitrogen (%)	0.90	0.86	0.97	1.02
Carbon (%)	79.46	86.90	70.38	60.75
Hydrogen (%)	0.30	0.41	0.44	0.75
Sulfur (%)	3.09	1.36	3.04	4.45
Oxygen (%)	0.70	0.97	8.79	16.06
Surface area (m^2^/g)	57	302	197	82
I_D_/I_G_	0.96	1.01	0.94	0.93

Note: Adapted from Tables 2 and 3 from Gonzalez-Gonzalez et al. [33].

**Table 2:** The atomic percentage of functional groups deconvoluted in C1s, N1s, and S2p from XPS spectrum of carbon dots samples.

Components	Atomic %			
	Cd.CB	Cd.H_2_SO_4_	Cd.KOH	Cd.H_2_SO_4_ + KOH
C 1s	39.31	52.23	56.38	58.45
C sp^2^	34.47	35.47	37.69	33.56
C sp^3^	41.18	33.05	24.81	25.66
C−N/C−S	4.25	5.26	10.9	8.82
Hydroxyl (C−OH)	5.99	4.70	4.04	3.01
Carboxyl (O−C=O)	14.22	10.79	22.56	19.67
Carbonyl (C=O)	-	9.82	-	6.89
Ether (C−O−C)	-	0.92	-	-
Carbonate (CO_3_^2–^)	-	-	-	2.39
N1s	0.82	1.78	1.8	2.28
Imide (O=C−N−C=O)	65.04	70.7	21.54	65.51
Nitro (NO_2_)	34.96	29.3	31.58	34.49
N−(C=O)−N	-	-	35.88	-
S2p	0.8	4.78	1.73	1.69
Sulfoxides (S=O)	69.02	28.18	-	-
Sulfonic acid (SO_3_H)	30.98	71.82	74.64	77.97
Sulfides (C−S−C)	-	-	25.36	22.03

**Table 3:** Fitting parameters from Raman spectra of carbon dots samples.

	Cd.CB	Cd.H_2_SO_4_	Cd.KOH	Cd.H_2_SO_4_ + KOH
I_D_/I_G_	1.13	1.30	1.38	0.99
D band position (cm^−1^)	1355	1355	1357	1341
D band area	153	219	121	156
D band height	0.92	0.88	0.79	0.93
D band FWHM	106	158	98	107
G band position (cm^−1^)	1596	1588	1602	1591
G band area	59	88	44	85
G band height	0.81	0.68	0.57	0.93
G band FWHM	106	82	49	58

**Table 4:** ζ Potential results from carbon dots samples.

Sample	ζ Potential (mV)			
Cd.CB	−11.61	±	1.59	
Cd.H_2_SO_4_	−5.20	±	2.60	A
Cd.KOH	−4.22	±	2.17	A
Cd.H_2_SO_4_ + KOH	−1.28	±	1.16	

Note: Mean values with the same letter are not statistically different (Tukey’s test, *p*-value > 0.05).

## Data Availability

The data presented in this study are available on request from the corresponding author.
